# Improving the Function and Engraftment of Transplanted Pancreatic Islets Using Pulsed Focused Ultrasound Therapy

**DOI:** 10.1038/s41598-019-49933-0

**Published:** 2019-09-16

**Authors:** Mehdi Razavi, Fengyang Zheng, Arsenii Telichko, Jing Wang, Gang Ren, Jeremy Dahl, Avnesh S. Thakor

**Affiliations:** 1 Interventional Regenerative Medicine and Imaging Laboratory, Stanford University School of Medicine, Department of Radiology, Palo Alto, California, 94304 USA; 2 Jeremy Dahl Ultrasound Laboratory, Stanford University School of Medicine, Department of Radiology, Palo Alto, California, 94304 USA; 3 Department of Ultrasound, Zhongshan Hospital, Fudan University and Shanghai Institute of Medical Imaging, Shanghai, 200032 China

**Keywords:** Type 1 diabetes, Animal disease models

## Abstract

This study demonstrates that pulsed focused ultrasound (pFUS) therapy can non-invasively enhance the function and engraftment of pancreatic islets following transplantation. *In vitro*, we show that islets treated with pFUS at low (peak negative pressure (PNP): 106kPa, spatial peak temporal peak intensity (I_sptp_): 0.71 W/cm^2^), medium (PNP: 150kPa, I_sptp_: 1.43 W/cm^2^) or high (PNP: 212kPa, I_sptp_: 2.86 W/cm^2^) acoustic intensities were stimulated resulting in an increase in their function (i.e. insulin secretion at low-intensity: 1.15 ± 0.17, medium-intensity: 2.02 ± 0.25, and high-intensity: 2.54 ± 0.38 fold increase when compared to control untreated islets; P < 0.05). Furthermore, we have shown that this improvement in islet function is a result of pFUS increasing the intracellular concentration of calcium (Ca^2+^) within islets which was also linked to pFUS increasing the resting membrane potential (*V*_*m*_) of islets. Following syngeneic renal sub-capsule islet transplantation in C57/B6 mice, pFUS (PNP: 2.9 MPa, I_sptp_: 895 W/cm^2^) improved the function of transplanted islets with diabetic animals rapidly re-establishing glycemic control. In addition, pFUS was able to enhance the engraftment by facilitating islet revascularization and reducing inflammation. Given a significant number of islets are lost immediately following transplantation, pFUS has the potential to be used in humans as a novel non-invasive therapy to facilitate islet function and engraftment, thereby improving the outcome of diabetic patients undergoing islet transplantation.

## Introduction

Type 1 diabetes (T1D) is a chronic autoimmune disease caused by the selective destruction of insulin producing β cells within pancreatic islets^1^. Currently, T1D affects 1.4 million people in the United States and 30 million people globally, and its incidence is increasing at an alarming rate^2^. In order for patients with T1D to maintain glucose homeostasis and prevent long-term complications of hyperglycemia, the current standard of care is daily self-administered injections of insulin^3^. However, this can only keep blood glucose levels within a broad range and cannot respond dynamically to second-by-second changes in blood glucose variability. Although whole pancreas transplantation is an effective approach to restore the physiological control of blood glucose levels without the need for exogenous insulin injections, it is a major surgical procedure and is rarely indicated as a treatment for T1D^4^. An alternative is islet transplantation, where islets are extracted from a donor pancreas and then minimally invasively administered into the liver of a diabetic patient. However, islets need to be harvested from a donor pancreas during which time their vascular connections are severed. Furthermore, compared to solid organ transplantation, islet transplantation is unusual in that a surgical vascular anastomosis is not created^5^. Hence, for islets to survive following engraftment, they need to rebuild their network of blood vessels, derived from the host microvascular bed, to ensure they receive an adequate supply of oxygen and nutrients; a process which takes 2–4 weeks. As a result, up to 60% of islets are lost within the first 2 weeks following transplantation, mainly due to hypoxia from an underdeveloped blood supply as well as the instant blood-mediated inflammatory reaction (IBMIR) towards islets^6^. Together, this reduces the number of viable islets which ultimately jeopardizes the long-term success of any islet transplant.

**Figure 1:**
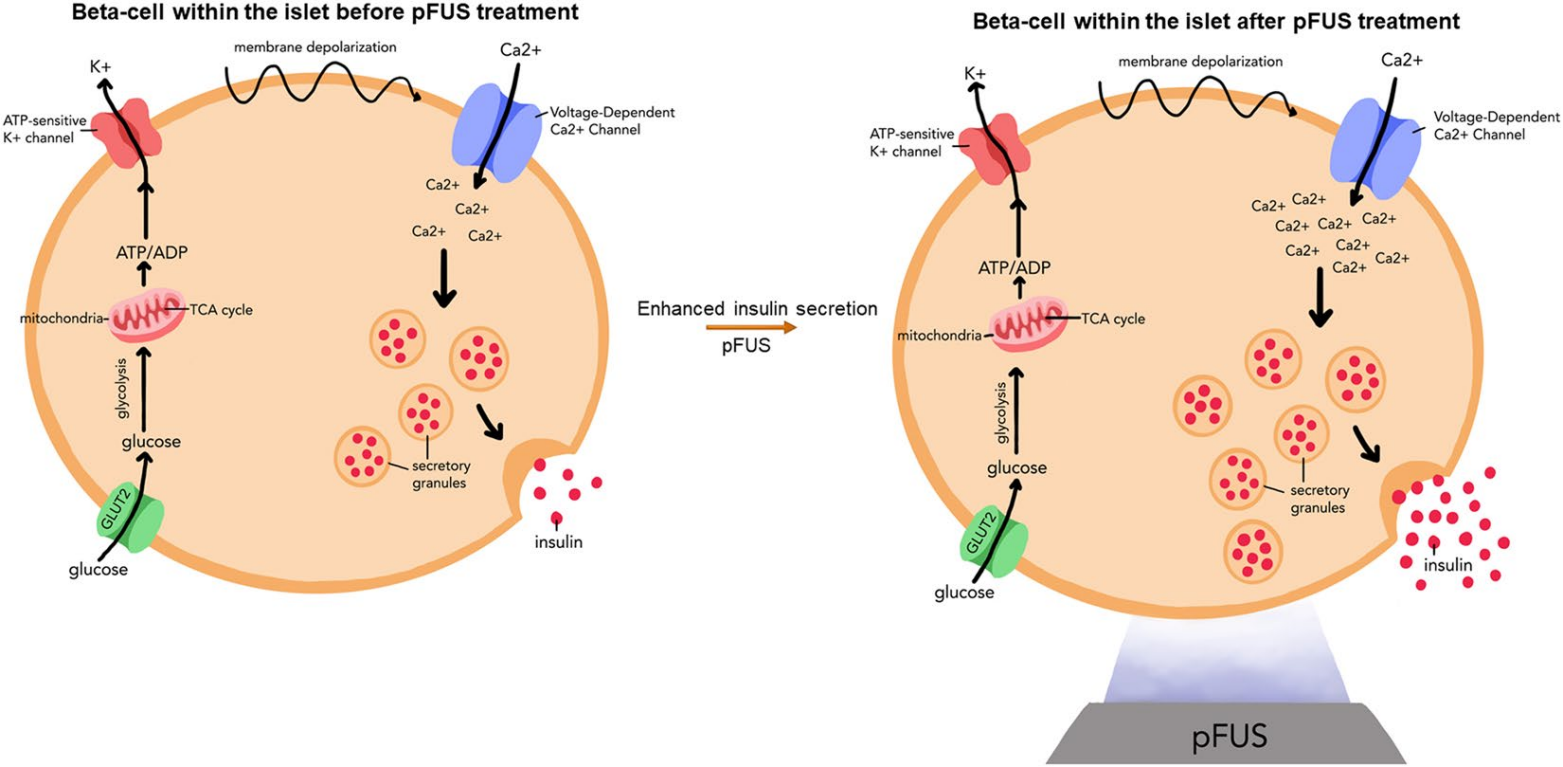
Schematic representation of how pFUS can stimulate insulin secretion: pFUS enhances insulin secretion from islets by enhancing intracellular calcium levels within β-cells which then triggers the release of insulin granules via exocytosis.

Once islets engraft following their transplantation, they then need to be able to function to release insulin from β cells. In response to elevated blood glucose levels, adenosine triphosphate (ATP)-sensitive potassium channels in β cells close, causing membrane depolarization thereby increasing intracellular free Ca^2+^ ([Ca^2+^]_i_). In turn, this triggers the exocytosis of insulin granules from β cells^7^ (Fig. 1). However, following islet transplantation, patients require immunosuppression therapy (i.e. tacrolimus and sirolimus), which has been shown to impair insulin secretion from islets^8,9^. Hence, a significant proportion of transplanted islets become “glucose-blind”, wherein β cells still contain insulin granules but cannot effectively release them in response to elevated glucose levels^10^. Interestingly, recent studies have shown that ultrasound, in certain conditions, can have several biological effects including an increase in calcium influx into cells^11^, including insulinoma β cells^10^. Furthermore, ultrasound has been shown *in vivo* to induce the formation of new blood vessels^12^. Hence, it is plausible that ultrasound could be used therapeutically in the setting of islet transplantation, not only to facilitate islet function, but also to help with islet engraftment and revascularization.

Currently, focused ultrasound (FUS) is used as a clinically-available modality that applies acoustic waves at specific locations within the body to induce a therapeutic effect without affecting overlying tissues^13,14^. For example, high intensity focused ultrasound (HIFU), a form of FUS, is now used to treat various conditions such as uterine fibroids, bone and prostate tumors by ablating the diseased tissue^15^. Pulsed focused ultrasound (pFUS) is a variation of this technology that uses short duty-cycles to minimize temperature elevations, thereby allowing the mechanical effects of ultrasound to predominate^16^. Hence, in the present study, we will explore whether pFUS can stimulate insulin secretion from pancreatic islets by increasing [Ca^2+^]_i_ within β cells. Next, we will examine *in vivo* whether applying pFUS to the site of islet transplantation can improve the function, viability and engraftment of transplanted islets within recipient diabetic animals. We will induce diabetes in mice using streptozocin (STZ) and then transplant islets under the renal capsule, which is a well-established technique for islet transplantation in small animal models^17^.

## Results

### *In Vitro* analysis of islet survival and function

**Figure 2:**
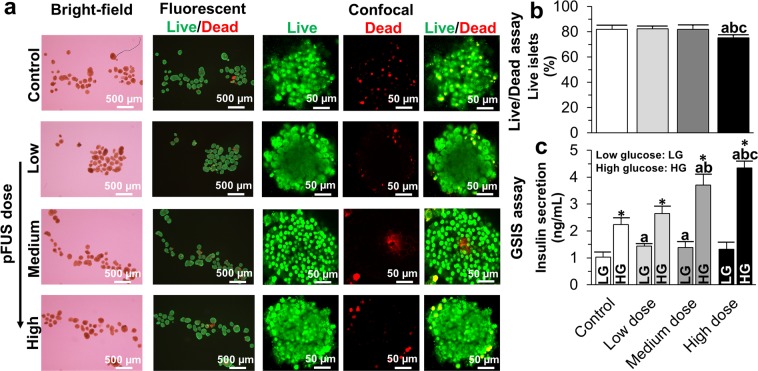
*In vitro* analysis of islet survival and function: (**a**) Bright-field, fluorescent and confocal images of control (i.e. non-pFUS treated islets) and islets treated with pFUS at low, medium and high acoustic intensities; (**b**) Results of live/dead assay; and (**c**) GSIS assay. Significant differences: ^a^P < 0.05: control *vs*. low or medium or high dose; ^b^P < 0.05: low *vs*. medium or high dose; ^c^P < 0.05: medium *vs*. high dose; *P < 0.05: low *vs*. high glucose (Two (**c**) or one (**b**)-way ANOVA post-hoc Tukey Test).

Following sonication with pFUS at low, medium and high intensities, islets maintained their spherical shape and kept their integrity, thereby confirming that pFUS does not adversely affect islet quality (Fig. 2a). Using a live/dead assay, the percentage of live islets in the control group (i.e. non-pFUS treated islets) was 82.00 ± 3.21% which was similar to islets treated with pFUS at low (82.33 ± 2.00%, P > 0.05) and medium (82.00 ± 3.05%, P > 0.05) intensities after 7 days of culture. However, there was a significant reduction in viability when islets were treated with pFUS at high intensities (Fig. 2b; 75.33 ± 2.08%, P < 0.05). Following stimulation of islets at both low and high glucose concentrations, the concentration of insulin secreted by islets significantly increased when pFUS was applied to islets, in an acoustic intensity-dependent manner: (Low glucose stimulation = control: 1.03 ± 0.18; low intensity: 1.44 ± 0.09; medium intensity: 1.38 ± 0.22; high intensity: 1.32 ± 0.26 ng/ml. High glucose stimulation = control: 1.23 ± 0.23; low intensity: 2.64 ± 0.27; medium intensity: 3.71 ± 0.40; high intensity: 4.35 ± 0.24 ng/ml) (Fig. 2c; P < 0.05).

**Figure 3:**
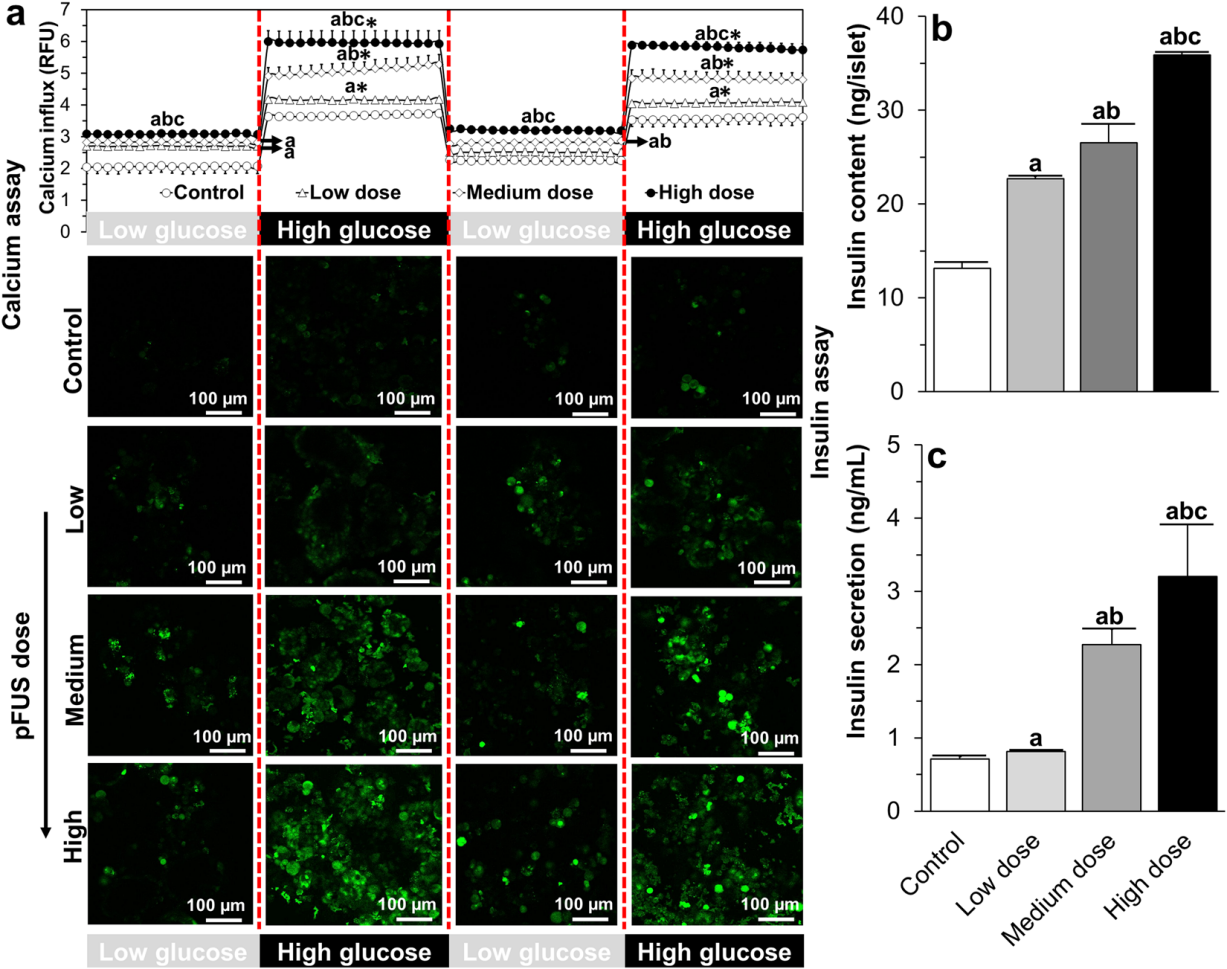
Calcium imaging and insulin assessment of islets: (**a**) Results of calcium imaging during 2 low-high glucose challenges and representative confocal images of the peak calcium response to glucose challenges; and (**b**) the insulin content within control (i.e. non-pFUS treated islets) and islets treated with pFUS at low, medium and high intensities and (**c**) the amount of insulin released from control (i.e. non-pFUS treated islets) and islets treated with pFUS at low, medium and high intensities. ^a^P < 0.05: control *vs*. low or medium or high dose; ^b^P < 0.05: low *vs*. medium or high dose; ^c^P < 0.05: medium *vs*. high dose; *P < 0.05: low *vs*. high glucose (Two (**a**) or one (**b**,**c**)-way ANOVA post-hoc Tukey Test).

Following glucose stimulation, there was an increase in fluorescence signal intensity in islets thereby indicating an increase in intracellular calcium concertation. In control non-pFUS treated islets, the fluorescence signal intensity was dependent on the amount of glucose present (Low glucose: 2.04 ± 0.21 *vs*. High glucose: 3.62 ± 0.07 relative fluorescence unit (RFU); p < 0.05) and this response pattern was maintained over two consecutive low-high glucose challenge cycles. Furthermore, this fluorescence signal intensity, and hence the amount of intracellular calcium within islets, was significantly increased at both low and high levels of glucose stimulation when islets were treated with pFUS in an acoustic-intensity dependent manner (Low glucose stimulation = control: 2.04 ± 0.21; low intensity: 2.70 ± 0.09; medium intensity: 2.82 ± 0.08; high intensity: 3.09 ± 0.04 RFU. High glucose stimulation = control: 3.62 ± 0.07; low intensity: 4.17 ± 0.0; medium intensity: 4.91 ± 0.26; high intensity: 5.99 ± 0.34 RFU; p < 0.05) (Fig. 3a). Under basal conditions (i.e. culture in RPMI medium at 37 °C and 5%CO_2_), control non-pFUS treated islets contained 13.14 ± 0.72 ng of insulin per islet and this concentration significantly increased when islets were treated with pFUS in an acoustic-intensity dependent manner: low intensity: 22.69 ± 0.24; medium intensity: 26.50 ± 2.03 and high intensity: 35.87 ± 0.33 ng (Fig. 3b; P < 0.05). Similarly, control non-pFUS treated islets secreted 0.71 ± 0.03 ng/ml of insulin into the surrounding medium and this concentration significantly increased when islets were treated with pFUS in an intensity-dependent manner: low intensity: 0.81 ± 0.02; medium intensity: 2.27 ± 0.21 and high intensity: 3.20 ± 0.71 ng/ml (Fig. 3c; P < 0.05).

**Figure 4:**
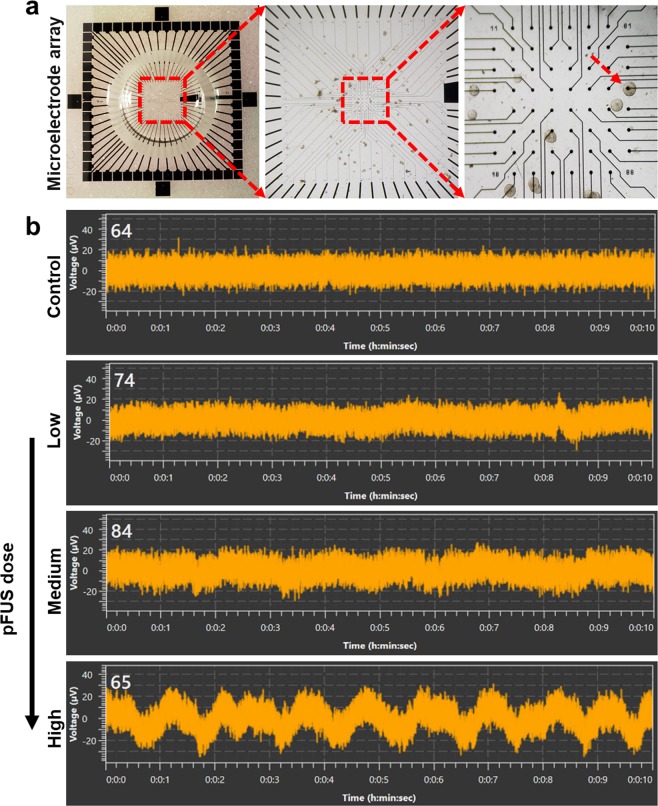
Recordings of membrane potential oscillations: (**a**) A microscopic view of a MEA; Islet placed on top of a MEA electrode (shown by arrow); (**b**) The characteristic pattern of the electrical activity in islets obtained at 16.7 mM glucose stimulation following pFUS treatment.

Under control conditions, islets showed no electrical activity (i.e. membrane potential oscillations), however, following pFUS stimulation there was an increase in electrical activity with a continuous spiking of membrane potential. Furthermore, our results showed that the membrane voltage of islets increased when they were treated with pFUS in an acoustic-intensity dependent manner (change in membrane voltage: control: 0; low intensity: 5 ± 2, medium intensity: 10 ± 3 and high intensity: 25 ± 5 μV, Fig. 4).

### *In Vivo* analysis of transplanted islets treated with pFUS

**Figure 5:**
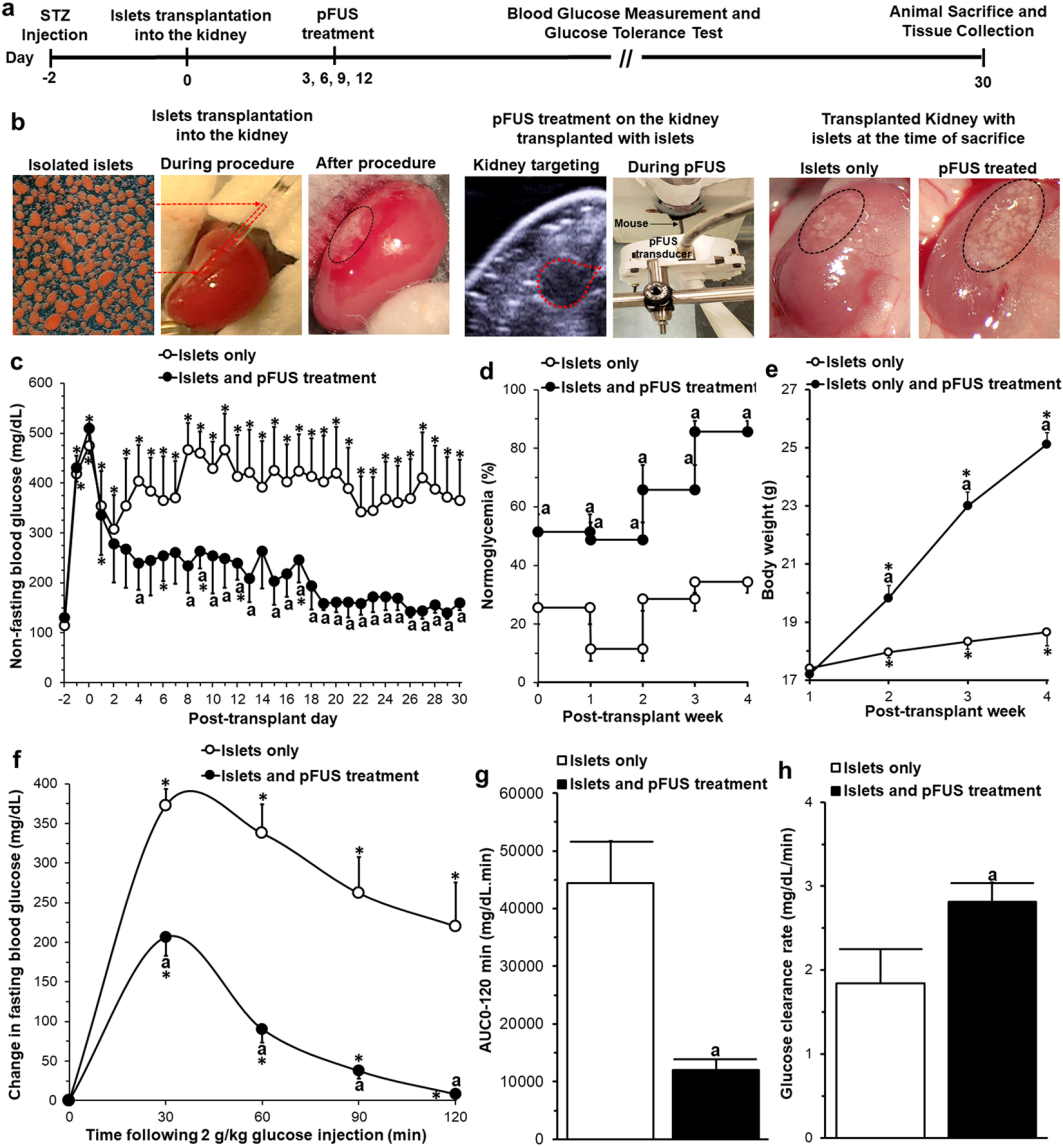
*In vivo* analysis following islet transplantation: (**a**) *In vivo* experimental details; (**b**) An example of an islet transplant (both control and islets treated with pFUS) using the kidney sub-capsule space as the site of transplantation: islets before, during and after transplantation (black circle = transplanted islets); pFUS treatment on the kidney containing the transplanted islets (red circle = site of pFUS targeting). Results of (**c**) blood glucose measurements, (**d**) normoglycemia percentage, (**e**) body weight, (**f**) IPGTT, (**g**) area under the IPGTT curve (AUC_0-120min_), and (**h**) blood glucose clearance rates calculated from the slope of IPGTT curves from 30 to 90 min. Significant differences: (**c**–**h**) ^a^P < 0.05: islets only *vs*. islets treated with pFUS; *P < 0.05: baseline *vs*. all other time-points (Two (**c**,**e**,**f**) or one (**b**)-way ANOVA with post-hoc Tukey Test or unpaired Student’s t-test (**g**,**h**)).

Experimental details of our *in vivo* experiment are outlined in Fig. 5a,b. Following *ip* injection of STZ, all mice became hyperglycemic with their non-fasting blood glucose values increasing from 122 ± 8 mg/dl (baseline, day −2) to 492 ± 17 mg/dl (post-STZ treatment, day 0). Following islet transplantation, both experimental groups showed a significant decrease in their non-fasting blood glucose levels within the first 48 h (islet transplantation alone: 307 ± 67 mg/dl and islet transplantation with pFUS treatment: 277 ± 76 mg/dl). However, after 48 h the blood glucose levels began to rise again in animals which were transplanted with islets alone, and this persisted for the duration of the experimental protocol (day 30: 364 ± 82 mg/dl). In contrast, in animals which were transplanted with islets that were then treated with pFUS, there was a continual drop in non-fasting blood glucose levels such that by the end of the experimental protocol all animals had become normoglycemic and had thus re-established glycemic control (day 30: 159 ± 15 mg/dL; Fig. 5c). Indeed, in this group of animals 51 ± 6% became normoglycemic in the first week post-transplantation with this number increasing to 86 ± 4% at week 4 post-transplantation (Fig. 5d). This was accompanied by mice progressively increasing their body weight from 17.2 ± 0.2 g (week 1) to 25.1 ± 0.4 g (week 4); an effect which was not seen in animals that were transplanted with islets alone which remained hyperglycemic throughout the experimental protocol (week 1: 17.4 ± 0.2 g to week 4: 18.7 ± 0.5 g; Fig. 5e).

In both experimental groups, blood glucose levels significantly increased following intraperitoneal glucose administration with a peak-value seen at 30 min at 2 weeks following transplantation (P < 0.05). However, in the animals treated with pFUS the change from baseline to the peak glucose value was significantly lower for the same glucose challenge (206 ± 23 vs 371 ± 23 mg/dL; p < 0.05) and by 120 min, these animals had restored their glucose values back to baseline levels unlike those animals which received islet transplantation alone (Fig. 5f). Accordingly, the AUC_0-120min_ (11,988 ± 1,881 vs 44,345 ± 7,315 mg/dl.min; Fig. 5g) was significantly lower and the glucose clearance rate (2.8 ± 0.2 vs 1.9 ± 0.4 mg/dl/min; Fig. 5h) was significantly faster in animals which had received islet transplantation and were treated with pFUS, compared to those animals which received islet transplantation alone (P < 0.05).

**Figure 6:**
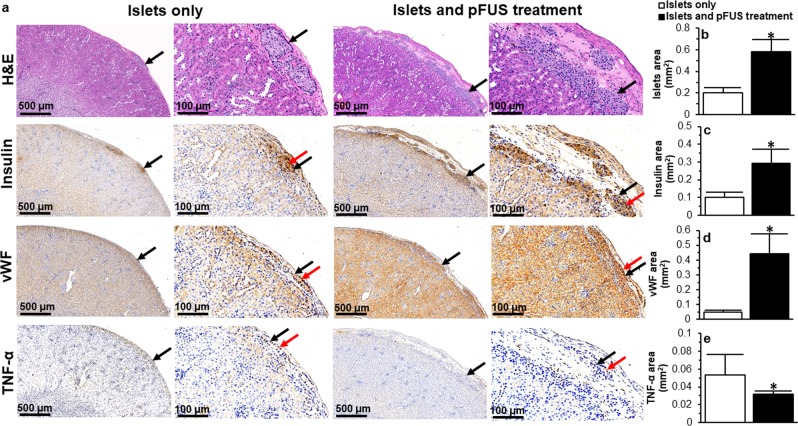
Histological assessment of transplanted islets: (**a**) Representative images following H&E, insulin, vWF and TNF-α immunohistochemical staining of islets that were transplanted under the kidney capsule. Black arrows = islets; Red arrow = positive (dark brown) staining; (**b**) Quantification of the surface area occupied by islets; (**c–e**) Quantification of positive (**c**) insulin, (**d**) vWF and (**e**) TNF-α staining. For all figures, control samples (non-treated islets) are compared to islets treated with pFUS. Significant differences: *P < 0.05 for islets only *vs*. islets treated with pFUS (Student’s unpaired t-test).

In animals treated with pFUS, there was a significantly greater number of viable transplanted islets in histological specimens compared to those animals which did not receive any pFUS treatment (total islet area: 0.58 ± 0.11 vs 0.20 ± 0.05 mm^2^, P < 0.05; Fig. 6a,b). Transplanted islets treated with pFUS retained their native size, spherical morphology, and maintained their intrinsic architecture with β cells (positive insulin staining) located in the center of the islets; findings which were not found in islets transplanted alone (Fig. 6a). There was a significant increase in insulin staining in pFUS treated islets compared to islets only (insulin-positive area per section: 0.28 ± 0.08 vs 0.10 ± 0.03 mm^2^; P < 0.05; Fig. 6a,c). Following treatment with pFUS, transplanted islets demonstrated a greater degree of vascularity. This was confirmed using immunohistochemical analysis which showed a significantly higher expression of vWF in pFUS treated islets (vWF-positive area per mm^2^ section: 0.44 ± 0.14 vs 0.05 ± 0.01; P < 0.05; Fig. 6a,d). Transplanted islets treated with pFUS also demonstrated reduced inflammation as evidenced by a reduction in the presence of TNF-α (TNF-α-positive area per section: 0.05 ± 0.02 vs 0.03 ± 0.003 mm^2^; P < 0.05; Fig. 6a,e).

**Figure 7:**
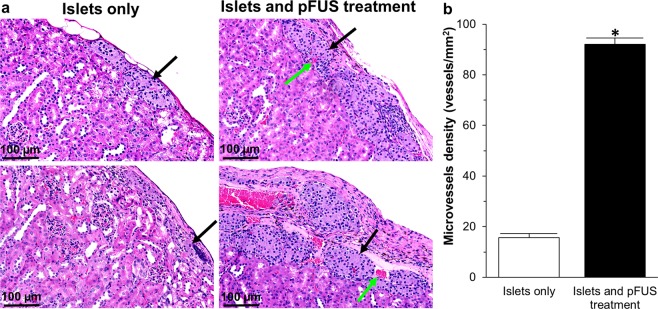
H&E histological assessment of transplanted islets: (**a**) Representative H&E histological staining of islets that were transplanted under the kidney capsule. Black arrows = islets; Green arrows = blood vessels; (**b**) Quantification of microvessel density within islets. In this figure, control samples (transplanted islets with no treatment) are compared to transplanted islets treated with pFUS. Significant differences: *P < 0.05 for transplanted islets only *vs*. islets treated with pFUS (Student’s unpaired t-test).

The greater degree of vascularity both surrounding, and within, transplanted islets treated with pFUS was further confirmed by H&E staining. Results showed significantly increased microvessel density for transplanted islets treated with pFUS compared to islets transplanted alone (92 ± 3 vs 16 ± 2 vessels/mm^2^; Fig. 7).

**Figure 8:**
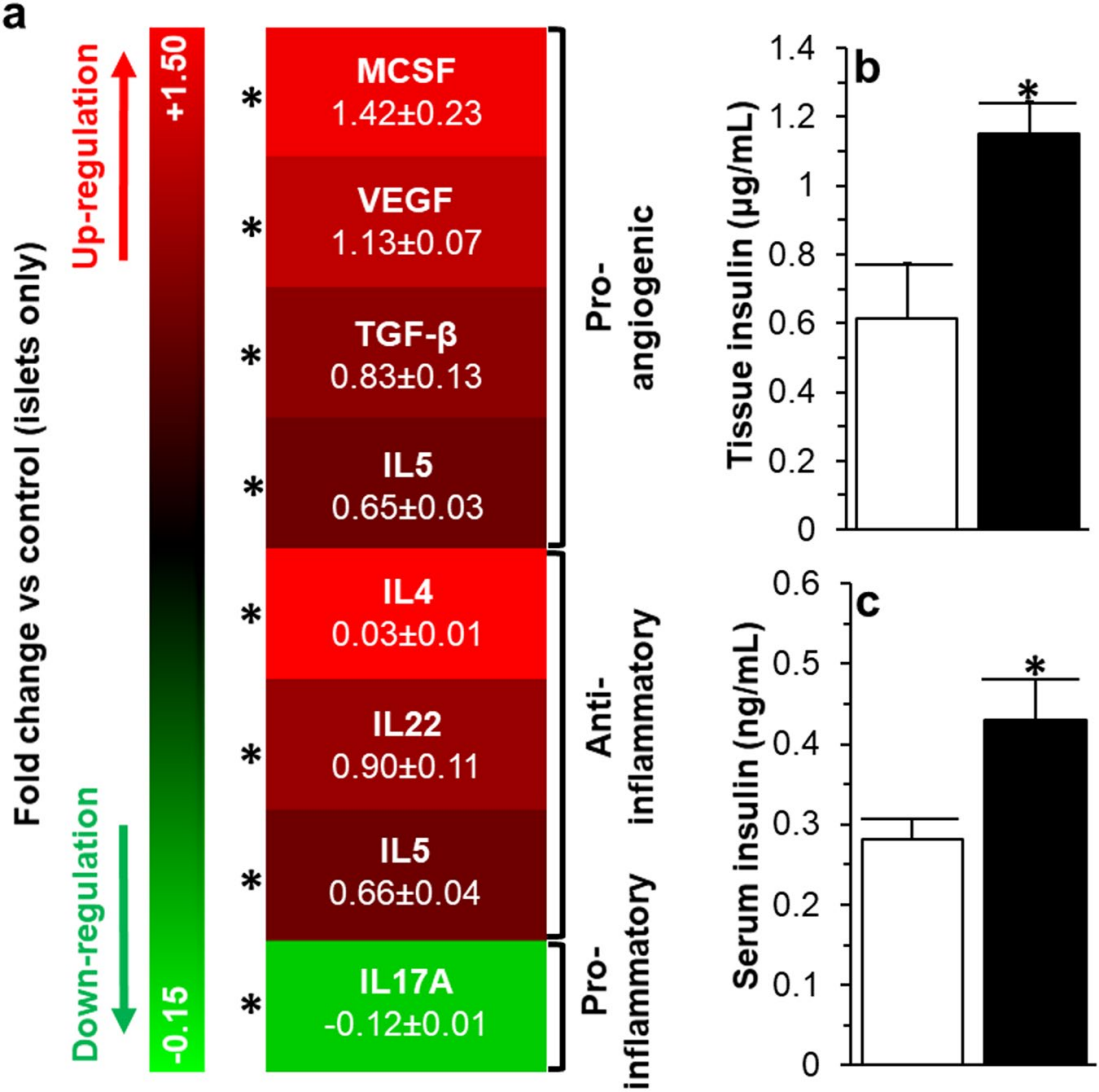
*Tissue and Blood analysis following islet transplantation*: (**a**) Cytokine expression profile and (**b**) insulin content within the kidney tissue of animals receiving an islet transplant. (**c**) Blood serum levels in animals receiving an islet transplant. For all figures, control samples (non-treated islets) are compared to islets treated with pFUS. Significant differences: *P < 0.05 for islets only *vs*. islets treated with pFUS (Student’s unpaired t-test).

Within the kidneys which contained the transplanted islets that were treated with pFUS, we also noted an up-regulation of macrophage colony-stimulating factor (MCSF: 1.42 ± 0.23 fold increase), vascular endothelial growth factor (VEGF: 1.13 ± 0.07 fold increase), transforming growth factor beta (TGF-β: 0.83 ± 0.13 fold increase), Interleukin 5 (IL5: 0.65 ± 0.03 fold increase), 4 (IL4: 0.03 ± 0.01 fold increase), 22 (IL22: 0.90 ± 0.11 fold increase), 5 (IL5: 0.66 ± 0.04 fold increase), and down-regulation of Interleukin 17 A (IL17A: 0.12 ± 0.01 fold decrease) when compared to the untreated kidneys which contained the transplanted islets alone (Fig. 8a; P < 0.05). Analysis of the explanted kidneys containing the islet transplant from animals treated with pFUS demonstrated a significantly higher amount of insulin compared with untreated animals (1.15 ± 0.09 vs 0.61 ± 0.16 μg/mL; P < 0.05; Fig. 8b). Similarly, the insulin content within the serum of animals treated with pFUS was significantly higher (0.43 ± 0.05 vs 0.28 ± 0.02 ng/ml; P < 0.05; Fig. 8c).

## Discussion

In the present work, we found that pFUS can (i) safely stimulate insulin secretion from islets via a voltage dependent mechanism which stimulated calcium influx into cells that was acoustic intensity dependent and (ii) be used *in vivo* to facilitate the function, engraftment and survival of transplanted islets by promoting islet revascularization as well as reducing inflammation. In the latter case, diabetic mice treated with pFUS not only demonstrated an improved ability to re-establish glycemic control, but they also showed a faster dynamic response to glucose challenges.

Although the discovery of insulin has changed the outlook and survival for diabetic patients for almost a century^18^, they are still not exempt from developing diabetic complications^19^. In part, this is due to the lack of tight regulation of glucose resulting in patients often having higher than normal blood glucose concentrations for sustained periods of time. Although islet transplantation aims to address this problem by establishing a functional islet mass in diabetic patients, it has encountered hurdles related to not enough islets surviving and engrafting following transplantation in addition to the surviving islets being able to function normally. Both of these issues can potentially be addressed with pFUS, which is a non-invasive technology that can target transplanted islets with acoustic waves, through imaging guidance. Indeed, our results show that when islets are treated with pFUS, their ability to release insulin from β cells in response to glucose is enhanced. Furthermore, this effect was acoustic intensity-dependent with higher intensities resulting in higher amounts of insulin released from islets. This is in keeping with studies from Castellanos *et al*. who also showed that ultrasound could be used to stimulate insulin from INS-1 pancreatic β cells^20^. However, in contrast to that study which showed that INS-1 pancreatic β cells were able to maintain their viability when stimulated with pFUS, we demonstrated that at high acoustic intensities (i.e. PNP of 212kPa and I_sptp_ of 2.86 W/cm^2^), there was a significant decrease in islet viability when compared to non-pFUS treated islets. This is likely due to the sensitive nature of islets compared to immortalized INS-1 pancreatic β cells, thereby potentially making them more susceptible to the mechanical effects of acoustic waves^21^. In addition, studies have shown that high ultrasound exposures can induce apoptosis in cells via mitochondria–caspase pathways and through inducing inertial cavitation^22^. Nevertheless, at medium intensities (i.e. PNP of 150 kPa, Isptp of 1.43 W/cm^2^), we observed an enhancement in islet function *in vitro*, with no decrease in viability.

Glucose enters β cells through the glucose transporter 2 (GLUT2) where it is then converted to pyruvate in the glycolysis pathway^23^. This results in an increase in the ATP/ADP ratio, which causes closure of ATP-sensitive K^+^ channels^24^ and thus membrane depolarization. In turn, this opens voltage-activated Ca^2+^ channels resulting in an influx of calcium, which then increases intracellular calcium ([Ca^2+^]_i_) that results in the release of insulin granules^23^. In the present study, when islets were treated with pFUS they demonstrated improved function; this can be attributed to pFUS-stimulating an increase in [Ca^2+^]i which subsequently can trigger insulin granule exocytosis^25,26^. This can be due to pFUS either (i) increasing resting membrane potential (*V*_*m*_) in β cells and hence reducing the threshold required to trigger depolarization and/or (ii) enhancing the influx of calcium following glucose stimulation. Furthermore, ultrasound has also been shown to stimulate calcium transients within cells^27^ as well as transiently induce cell membrane permeabilization by creating re-sealable pores on cell membranes as a result of acoustic cavitation (both stable and inertial)^28,29^. Together, these effects can enable ions (including Ca^2+^) to enter into β cells resulting in membrane depolarization and insulin secretion^27^. Future studies will aim to determine the relative contributions of each of the above effects in facilitating the Ca^2+^-dependent enhancement in insulin release following pFUS observed in the present study.

When 175 islets alone were transplanted into diabetic animals, hyperglycemia could not be reversed; this is in keeping with other studies which have shown similar results using this sub-therapeutic number of islets alone^30,31^. However, when islets were sonicated with pFUS over the first 2 weeks following transplantation, diabetic animals could now re-establish glycemic control which was sustained for 30 days following transplantation. In addition, these animals also showed faster dynamic responses to glucose challenges compared to animals that were not treated with pFUS.

Elevated levels of glucose in the body (glucotoxicity) have been shown to contribute to the worsening functioning of both native and transplanted islets^32^. Here, we demonstrated how pFUS can change the insulin content within islets as well their ability to release insulin in response to glucose. The improvement in islet function observed in this study could be attributed to pFUS stimulating insulin secretion from transplanted islets; in turn, this will ensure that the blood glucose levels within the body are maintained within normal limits thereby facilitating islet engraftment and function^33^. Histological examination of the islet graft after 1 month demonstrated that islets treated with pFUS had better morphology (insulin staining noted within the center of islets), enhanced vascularization (increased vWF staining and microvessel density on H&E staining) and reduced evidence of inflammation (decrease in TNF-α staining). Previous studies have shown that the architecture, organization and morphology of islets play a crucial role in their function and outcome following transplantation. When islets aggregate, the diffusion of oxygen and nutrients to cells within the center of larger aggregates will be limited compared to smaller aggregates or separated islets, thereby affecting their function and ultimately their survival^34^. Furthermore, the revascularization of smaller islets (and hence aggregates) has also been shown to be more efficient when compared to larger islets^35^.

Following islet transplantation, islets need to rapidly re-establish their vascular supply to ensure that they receive an adequate supply of nutrients and oxygen for survival^36^. If their revascularization is either delayed or is insufficient, islets will not survive and this will ultimately affect the overall function of the transplant^37,38^. Given that von Willebrand Factor (vWF) acts as a regulator of angiogenesis by controlling vessel proliferation and maturation^39^, our data shows that pFUS increases vWF immunoreactivity in transplanted islets suggesting that soundwaves can help to promote vessel proliferation and maturation, thereby helping islets to form a more functional microcirculation. In addition, within the kidneys containing the transplanted islets, we also found the following pro-angiogenic factors to be upregulated: MCSF^40^, VEGF-A^41^, TGF-β^42^, and IL5^43^; Studies have shown that MCSF induces monocytes to produce and release VEGF-A which promotes endothelial cell (EC) proliferation and new blood vessel formation^40^. In addition, multiple studies have shown that VEGF-A is crucial for the revascularization of islets following transplantation^44^ and β-cells themselves have been shown to secrete large amounts of VEGF-A, which is mitogenic for ECs and crucial for maintaining the density and specialty phenotype of fenestrated intra-islet ECs^45^. Similarly, TGF-β has been shown to enhance islet survival and function by inducing islet neogenesis^46^ as well as promoting EC survival during angiogenesis^42^. While the role of IL-5 is less well defined, studies have shown that it has both pro-angiogenic^43^ and anti-inflammatory^47^ attributes. Hence, the observed increase in pro-angiogenic factors within the kidneys containing transplanted islets can potentially be attributed to these factors being secreted by islets following their treatment with pFUS; this is further supported by improved vascularization of transplanted islet as demonstrated by histological (i.e. H&E) and immunohistochemical (i.e. vWF staining) analysis of tissue samples. Taken together, is therefore plausible that pFUS is able to increase the release of these factors either indirectly (via its effect on the engraftment site – i.e. kidney) or directly (via stimulating individual cells within the transplanted islets).

In addition to the pro-angiogenic factors that were upregulated, pFUS also increased other cytokines such as TGF-β^48^, IL4^49^, IL22^48^, and IL5^50^, which have been shown to have anti-inflammatory properties, while decreasing cytokines such as IL17A^51^, which have been shown to have pro-inflammatory properties. In keeping with this, we also observed a reduction in TNF-α staining within the transplanted islets that were treated with pFUS. This is important as the inflammatory response mounted by the recipient to transplanted islets has been shown to play a significant role in poor islet engraftment and survival^52^.

The clinical translation of this pFUS to T1D patients is relatively feasible given that the equipment necessary to accomplish pFUS is identical to that used for HIFU treatments. Thus, it is only necessary to adjust the acoustic parameters to achieve the PNPs and acoustic intensities reported here. However, it should be noted that the PNPs and intensities measured here are non-derated values because the coupling medium (water) is non-attenuating and the depth at which the pFUS was applied in the animals was non-significant for the frequency utilized. For humans, it will be necessary to utilize acoustic parameters that achieve the reported PNPs and intensities after deration. Derating the PNPs and intensities will be necessary because, for clinical treatment, the transducer will be coupled directly to the individual (via acoustic coupling gel) and the acoustic pressure and intensities will be attenuated by the intervening tissue between the transducer and the target tissue region.

In summary, our results show that pFUS is safe and can stimulate the function of islets, via a Ca^2+^-dependent mechanism. Furthermore, pFUS can enhance the engraftment (through facilitating islet revascularization and reducing inflammation), function and survival of islets following transplantation. Given that FUS is an FDA approved technology, pFUS therefore has the potential to be easily clinically translated as a completely non-invasive and drug-free therapeutic approach which can be utilized in the setting of islet transplantation.

## Methods

### Isolation and culture of islets

Pancreatic islets were isolated from C57/B6 mice (male, 6–8 week-old, Charles River Laboratories, USA), as previously described (see *Supplemental Information*)^53^.

### *In Vitro* treatment of islets with pFUS

Details of the pFUS set-up, calibration and output characterizations are described in the Supplemental Information and Supplemental Fig. 1. For each pFUS treatment, experiments were performed using a 12 well-plate (Corning, USA) containing 100 islets/well. Given that the ultrasound beam width (16 mm) was close to the diameter of an individual well, this allowed the simultaneous sonication of all the islets since they were predominantly seeded in the center of a well. Ultrasound gel was applied on the surface of the piston transducer to couple it with the bottom of the well plate. For the *in vitro* experiments, the transducer was used with the following parameters: 1 MHz, 2000 cycle, sinusoidal pulses at a pulse repetition frequency (PRF) of 100 Hz for a 20% duty cycle (DC) and voltages of 12, 16.5, and 23.2 Vpk-pk to achieve three different acoustic intensities: low, medium and high, with a total pFUS exposure time of 1 min (Table 1). The selection of pFUS parameters was based on previous literature showing that these parameters could improve cellular function with no adverse effect on cell growth and/or viability^20,54^.

**Table 1:** Acoustic Output of pFUS used for *in vitro* treatment of islets*.

Acoustic Output	DC (%)	PRF (Hz)	PNP (kPa)	MI (MPa/MHz^1/2^)	TI (°C)	Isata (W/cm^2^)	Isapa (W/cm^2^)	Isptp (W/cm^2^)	Time (min)
Low	20	100	106	0.11	0.37	0.05	0.14	0.71	1
Medium	20	100	150	0.15	0.74	0.10	0.27	1.43	1
High	20	100	212	0.21	1.48	0.20	0.55	2.86	1

### *In Vitro* analysis of Islets treated with pFUS

Islet viability, glucose stimulated insulin secretion (GSIS) assay, calcium assay, and recording of membrane potential oscillations were performed as described in the Supplemental Information. All experiments were performed in triplicate where each individual experiment contained 30 islets in a 96 well plate (30 islets/well). As required, pFUS treated islets were selected from a 12 well-plate which contained 100 islets per well. There were 4 experimental groups tested: Group 1 = no pFUS stimulation of islets (control); Group 2 = islets stimulated with pFUS at a low intensity (PNP: 106 kPa, I_sptp_: 0.71 W/cm^2^); Group 3 = islets stimulated with pFUS at a medium intensity (PNP: 150 kPa, I_sptp_: 1.43 W/cm^2^); and Group 4 = islets stimulated with pFUS at a high intensity (PNP: 212 kPa, I_sptp_: 2.86 W/cm^2^).

### Islet transplantation and treatment with pFUS

All experiments were approved by the Institutional Animal Care and Use Committee (IACUC) at Stanford University and all experiments were performed in accordance with relevant guidelines and regulations. Male C57BL/6 mice, at 6–8 weeks age (Charles River Laboratories, USA), were used as both donors and recipients. All animals were maintained on a 12 h:12 h light:dark cycle with *ad libitum* access to food and water. Recipient mice were matched for their body weight and baseline blood glucose levels and then randomly assigned into 2 experimental groups: Group 1: mice transplanted with islets only (n = 5; Control Group) and Group 2: mice transplanted with islets followed by treatment with pFUS at days 3, 6, 9, and 12 post-transplantation (n = 5). Prior to islet transplantation, all recipient mice were made diabetic (i.e. determined by 2 consecutive non-fasting blood glucose levels > 350 mg/dl, as previously documented^55^) by an intraperitoneal injection of streptozotocin (STZ; 180 mg/kg). Isolated islets from C57BL/6 mice were cultured overnight before transplantation to allow the islets to rest following the isolation procedure prior to being transplanted as well as to enable quality control testing of the islets. Each diabetic mouse then received 175 handpicked islets, which were implanted under the right kidney capsule, before being randomly allocated to an experimental group.

Islets were then treated with pFUS as described in the Supplemental Information. To treat the whole kidney, 8 non-overlapping adjacent regions through the kidney were targeted for 30 sec per region. The time to treat one kidney with these parameters was approximately 4 min. In order to deliver pFUS therapy to the animal, the HIFU transducer was used with the following parameters: 5% DC, 5 Hz PRF, 2.9 MPa PNP, and 272 W/cm^2^ I_sapa_, which has been shown in previous studies to be safe in small animals (Table 2)^56^. After pFUS treatment, each mouse was removed from the water bath, dried, and placed in a recovery cage.

**Table 2:** Acoustic Output of pFUS used for *in vivo* treatment of islets*.

	DC (%)	PRF (Hz)	PNP (MPa)	MI (MPa/MHz^1/2^)	TI (°C)	I_sata_ (W/cm^2^)	I_sapa_ (W/cm^2^)	I_sptp_ (W/cm^2^)	Time (min)
Acoustic Output	5	5	2.9	2.8	1.6	13	272	895	4

*The values presented in Tables 1 and 2 are non-derated values.

DC: Duty Cycle.

PRF: Pulse Repetition Frequency.

PNP: Peak Negative Pressure.

MI: Mechanical Index at 1.1 MHz.

TI: Thermal Index.

I_sata_: Spatial Average Temporal Average Intensity.

I_sapa_: Spatial Average Pulse Average Intensity.

I_sptp_: Spatial Peak Temporal Peak Intensity.

### Statistical analysis

All values were expressed as the mean ± standard error of the mean (SEM). Statistical analysis of all quantitative data was performed using a one or two-way ANOVA (Analysis of Variance) with post-hoc Tukey test (Astatsa.com; Online Web Statistical Calculators, USA) or unpaired Student’s t-test with any differences considered statistically significant when P < 0.05.

## Supplementary information

Supplementary Info

## Data Availability

All data supporting findings of this study are available within the article or from the corresponding author upon request.
