# Supplementary material for: Improving the Function and Engraftment of Transplanted Pancreatic Islets Using Pulsed Focused Ultrasound Therapy
Source: Sci Rep. 2019 Sep 16;9:13416. doi: 10.1038/s41598-019-49933-0 (PMC6746980; doi:10.1038/s41598-019-49933-0)
Supplement: Supplementary file 1 — Supplementary Info [file 41598_2019_49933_MOESM1_ESM.docx]

**SUPPLEMENTAL MATERIAL**

**Title:** IMPROVING THE FUNCTION AND ENGRAFTMENT OF TRANSPLANTED PANCREATIC ISLETS USING PULSED FOCUSED ULTRASOUND THERAPY

**Authors:** MEHDI RAZAVI^1^, FENGYANG ZHENG^1,3^, ARSENII TELICHKO^2^, JING WANG^1^, GANG REN^1^, JEREMY DAHL^2^ & ^*^AVNESH S THAKOR^1^

**Affiliations:** ^1^Interventional Regenerative Medicine and Imaging Laboratory, Stanford University School of Medicine, Department of Radiology, Palo Alto, California 94304, USA

^2^Jeremy Dahl Ultrasound Laboratory, Stanford University School of Medicine, Department of Radiology, Palo Alto, California 94304, USA

^3^Department of Ultrasound, Zhongshan Hospital, Fudan University and Shanghai Institute of Medical Imaging, Shanghai 200032, China

**Journal:** Scientific Reports

**Article Type:** Original Article

**Key Words:** Pulsed Focused Ultrasound; Therapeutic Ultrasound; Acoustic waves; Islet Transplantation; Diabetes.

**Corresponding Author:** *Avnesh S. Thakor, MD PhD

3155 Porter Drive

Department of Radiology, Stanford University

Palo Alto, CA, 94304

E-mail: [asthakor@stanford.edu](mailto:asthakor@stanford.edu)

Tel: 650-723-8061

Fax: 650-736-8937

**METHODS**

**Islet Isolation**

The common bile duct was cannulated with a 30G needle and the pancreas distended with 3ml of cold collagenase solution (Fischer Scientific, USA) ^1^. Islets were first isolated from the pancreas by digesting the gland at 37°C for 10min, and then purified using histopaque-density gradients. Islets were then washed with Hank's balanced salt solution (HBSS, Gibco, USA) supplemented with 0.1% bovine serum albumin (BSA, Gibco, USA) before being cultured in complete medium containing Roswell Park Memorial Institute (RPMI; Gibco, USA) medium supplemented with 10% fetal bovine serum (FBS; Invitrogen, USA) and 50U/mL penicillin-50µg/mL streptomycin in a humidified incubator at 37°C and 5% CO_2_.

***In Vitro* Treatment of Islets with pFUS**

1. **Set-up:** pFUS was performed on islets using a custom built system consisting of a function generator (33250A, Agilent, Santa Clara, CA), a power amplifier (ENI 525LA, Electronics & Innovation, Rochester, NY) and a focused piston transducer (2.54cm diameter, 5cm focal depth) operating at 1MHz center frequency.
2. **Output Characterization:** To measure the acoustic pressure and intensities for *in vitro* experiments, the transducer was submerged in a water tank containing degassed water and driven by sinusoidal ultrasound pulses at 1MHz frequency. The pulses were transmitted at a pulse repetition frequency (PRF) of 100Hz and utilized 5 cycles per pulse. A needle hydrophone (HNR0500, Onda Corporation, Sunnyvale, CA) was placed in front of the transducer and connected to an oscilloscope to measure the emitted acoustic waves. An Acoustic Intensity Measurement System (AIMS III, Onda Corporation., Sunnyvale, CA) was used to perform a raster scan of the pressure field with the hydrophone in the x-y plane 3 mm from the transducer surface. Digitized pressure waveforms were recorded with an oscilloscope (Agilent DSO6012a, Santa Clara, CA). A pressure field scan was performed for applied amplified voltages of 12, 16.5, and 23.2 Vpk-pk. The peak negative pressure (PNP) and intensities for each pressure field scan were computed for each voltage. The spatial-average temporal average intensity (I_sata_), spatial-average pulse-average intensity (I_sapa_), and the spatial-peak temporal-peak intensity (I_sptp_) were calculated by linearly scaling the pulse to 2000 cycles (i.e. the actual pulse length utilized in the *in vitro* experiments). A second set of pressure field scans was performed by inserting a polystyrene 12-well plate between the hydrophone and transducer, with the transducer position underneath a single well filled with degassed water (2 ml). The transducer was coupled to the plate using ultrasound gel (Aquasonic, Bio-Medical Instruments, USA). The hydrophone was submerged into the center of the well, 3mm away from the transducer, and pressure field scans were then obtained using the 3 voltages described previously (**Supplemental Figure 1**).


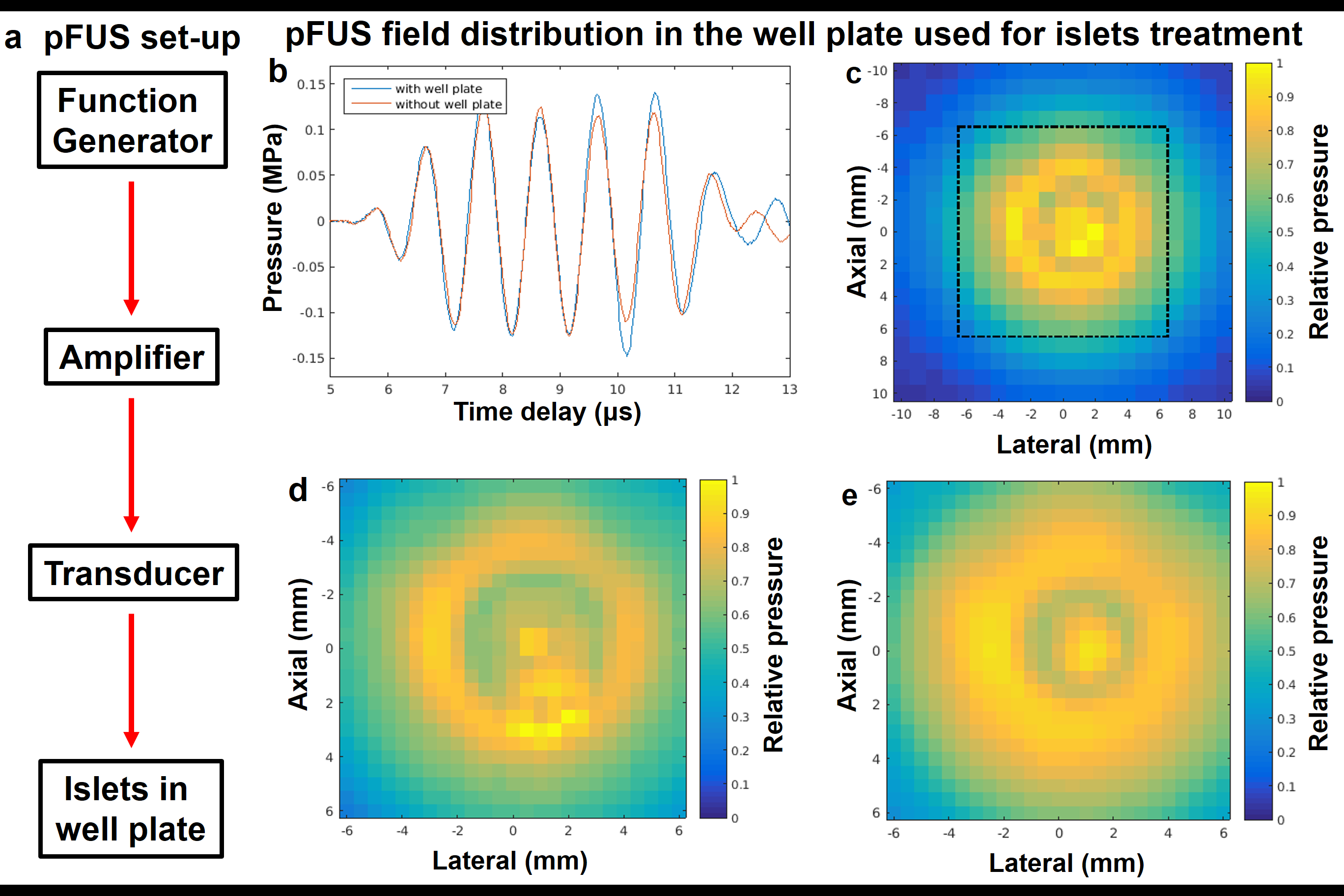


**Supplemental Figure 1. Summary of our *In vitro* pFUS set-up:** **(a)** pFUS set-up: the transmitted ultrasound waves are produced by the function generator, amplified through the amplifier at a constant gain and emitted from the transducer face; **(b-e)** Beam plots measured at 1MHz excitation and 3mm axially from the transducer: (b) received waveforms profiles with and without the presence of a well plate; (c) 2D relative peak-to-peak pressure scan in water without the presence of a well plate; (d) zoomed central region without the well plate and (e) zoomed central region with the well plate.

***In Vitro* Analysis of Islets Treated with pFUS**

1. **Islet Viability:** Cell viability was performed using a Live/Dead assay [i.e. fluorescein diacetate (FDA; for live cells, Thermofisher Scientific, USA) and propidium iodide (PI; for dead cells, Thermofisher Scientific, USA)] to assess the safety of our chosen pFUS parameters. Hence, following removal of any culture medium, islets were stained with a Live/Dead staining solution [FDA (75µL/well) and PI (75µL/well)] which was added and incubated with islets for 20min at 37°C. Next, the staining solution was removed and cells were washed three times with PBS. A live cell imaging solution (Thermofisher Scientific, USA) was then added to each well before imaging. Images were acquired with a Zeiss LSM710 Confocal Microscope at a magnification of 20X and figures created and analyzed with FIJI software (ImageJ, GNU General Public License).
2. **Glucose Stimulated Insulin Secretion (GSIS) Assay:** The ability of islets to secrete insulin was assessed by exposing them to low and high glucose media. In brief, islets were incubated in Krebs Ringer Buffer (KRB; Sigma-Aldrich, USA) spiked with 2.8mM glucose (low) for 2h followed by 16.7mM glucose (high) for 2h at 37°C at 5% CO_2_. The insulin content within the KRB medium was then quantified using a mouse insulin ELISA kit following the manufacturer's protocol (Mercodia, USA).
3. **Calcium Assay:** To assess intracellular levels of calcium following treatment with pFUS, calcium imaging was performed. Islets were loaded with an indicator, Fluo-4 (100μL/well; Sigma Aldrich, USA) for 45min, which fluoresces upon binding Ca^2+^ ^2^. Islets were then imaged using a Zeiss LSM710 Confocal Microscope at a magnification of 10X while being stimulated with a series of sequential glucose challenges using low (2.8mM) and high (16.7mM) glucose concentrations in KRB, before and after treatment with pFUS. Seventeen images were collected during each 5min glucose challenge to generate image stacks that were used to measure the cumulative Ca^2+^ signal over time. Quantification of the mean fluorescent intensity in the time series confocal images was performed using FIJI software (ImageJ, GNU General Public License).
4. **Insulin Assays:** The insulin content was measured both (i) within islets and (ii) in the solution in which islets were cultured (i.e. the amount secreted by islets). After each pFUS stimulation, both the RPMI medium and the islets themselves were collected for analysis. Islets were lysed using an acid-ethanol (0.18M hydrochloric acid in 96% ethanol) extraction protocol ^4^; in brief, islets were added to a lysis buffer contained Tris-hydrochloric acid (1mM, 500μL, pH 7.5, Sigma Aldrich, USA), ethylenediaminetetraacetic acid (EDTA; 0.5M, 500μL, pH 8, Sigma Aldrich, USA), bovine serum albumin (BSA; 50mg, Sigma Aldrich, USA) and deionized water (49mL). The islets were then added to an acid-ethanol extraction solution (lysis buffer/acid-ethanol extraction solution: 1/4 (v/v)). The insulin content within both the RPMI medium and extraction solution was then quantified using a mouse insulin ELISA kit following the manufacturer's protocol (Mercodia, USA).
5. **Recording of Membrane Potential Oscillations:** Extracellular membrane potential recordings were obtained using a microelectrode array (MEA; USB-1060 system and Multi-Channel Experimenter software; Smart-Ephys, USA) ^5^ which contained titanium-nitride electrodes with a diameter of 30μm (200/30-Ti; Multi Channel Systems). The data was acquired using a low-pass filter at 100Hz and sampled at 10kHz. Islets, with a diameter of 150μm, were placed on the top of electrodes using a pipette at 37°C and 5% CO_2_. Extracellular voltage changes were then recorded from the electrodes which had the islets (i.e. untreated and pFUS treated) following the addition of 16.7mM glucose into the medium using the grounded bath electrode as a reference. Islets from the MEA system were then transferred to a chamber at 37°C where their oscillatory activity was recorded.

***In Vivo*** **Treatment of Islets with pFUS**

1. **Set-up:** pFUS was administered to animals using a modified HIFU transducer (H-102NRE, Sonic Concepts, USA). This transducer has a 1.1MHz center frequency, a focal depth of 55mm, a 64mm outer diameter and a 49mm central opening to accommodate a diagnostic ultrasound imaging transducer. The transducer was driven by a function generator through a power amplifier, and had an impedance matching circuit (Sonic Concepts, USA). A diagnostic ultrasound transducer (14L5SP) connected to a Siemens ACUSON S2000 unit (Siemens Healthcare, Issaquah, WA) was utilized for guiding/targeting the pFUS treatment at the site of the islet transplantation.
2. **Calibration:** The HIFU transducer setup for acoustic output measurements was similar to the piston transducer calibration, except a fiber-optic hydrophone (FOH) (Precision Acoustics, Dorchester, UK) was used due to its ability to sustain high pressures induced by the HIFU transducer. The transducer was driven at 1.1MHz with 20 cycle bursts at a PRF of 100Hz (0.18% DC). The FOH hydrophone was placed at the focal spot of the transducer and the pressure fields and intensities were measured as previously described. The measured beam width (FWHM) at the focal zone was 1.5mm in diameter for a length of 10mm. The measured PNPs and intensities were then scaled to the desired PRF and DC.

1. **Image Guidance:** For precise targeting, the imaging transducer was mechanically coupled to the HIFU transducer using a custom 3D-printed holder that enabled the focal spot of the ultrasound therapy beam to lie in the ultrasound imaging plane. The focal spot of the HIFU transducer was fixed at 55mm axial and 0mm lateral distance from the center point of the imaging transducer. The AIMS III system, hydrophone, oscilloscope and Siemens ACUSON S2000 unit were used to align the ultrasound beam and imaging plane to within a tolerance of 200μm. Mice that had received an islet transplant were first anesthetized and then inserted vertically, up to the level of their neck, into a water bath containing heated (37°C) degassed water to couple the ultrasound to the animal. The assembled holder with the HIFU transducer was then attached to a translation stage to enable translation of the focal spot and to keep it fixed during the pFUS therapy. Using real-time ultrasound imaging guidance, the mouse was positioned so the right kidney was in the center of the focal point of the HIFU transducer.

***In Vivo* Analysis of Transplanted Islets Treated with pFUS**

1. **Metabolic Analysis:** All metabolic analyses were performed in conscious, restrained mice at the indicated time points. For all tests, blood glucose was measured via tail vein sampling using a handheld glucometer (Bayer Contour Glucose Meter, USA). Mice were considered normoglycemic when non-fasting blood glucose levels were <200mg/dl ^6^. Intraperitoneal glucose tolerance tests (IPGTT) were performed at week 2 post-transplantation after overnight fasting and an injection of glucose (2g/kg). Blood glucose values were then measured at indicated time points allowing for the area under the curve (AUC) and blood glucose clearance rate to be calculated between transplantation groups.

At euthanasia, the kidneys containing the transplanted islets that had been treated with or without pFUS were harvested, and then proceed for either histological analysis (i.e. fixed in 4% paraformaldehyde (PFA), dehydrated with graded ethanol solutions, embedded in paraffin and sliced with a microtome) or for molecular analysis (i.e. tissues were stored at -80°C for subsequent processing).

1. **Histological Analysis:** Sections were prepared for histological and immunohistochemical analyses to determine islet structure and viability (Haemotoxylin and Eosin (H&E) and insulin staining), evidence of vascularization (H&E and von Willebrand factor (vWF) staining) and inflammation (H&E and tumor necrosis factor alpha (TNF-α) staining) via standard procedures. The stained sections were then imaged using a NanoZoomer slide scanner 2.0-RS (Hamamatsu, Japan). Results were analyzed using FIJI Image J software with at least 15-20 islets from 5 different sections through the kidney of each animal.
2. **Molecular Analysis:** At euthanasia, blood samples were also collected to measure the serum insulin levels (insulin ELISA kit; Mercodia). The frozen kidney tissue was then homogenized as follow: tissue samples were placed in a homogenization buffer at a ratio of 1 kidney/1mL buffer; the buffer contained a protease inhibitor combination (Sigma Aldrich, USA) including 4-(2-Aminoethyl)benzenesulfonyl fluoride hydrochloride (AEBSF, 2mM), Aprotinin (0.3μM), Bestatin (116μM), trans-Epoxysuccinyl-L-leucylamido(4-guanidino)butane (E-64, 14μM), Leupeptin (1μM) and ethylenediaminetetraacetic acid (EDTA, 1mM) in tissue protein extraction reagent (ThermoFisher Scientific, USA) containing phenylmethylsulfonyl fluoride (PMSF). All homogenized kidney samples were sonicated 3 times for a total of 8s (Branson SLPe) and then placed on a rotisserie at 4°C for 45min before being centrifuged at 4°C, 15000rpm for 15min. The tissue supernatant was then collected and the insulin content measured (mouse insulin ELISA kit; Mercodia) as well as the level of tissue cytokines (mouse multiplex ELISA; eBiosciences/Affymetrix/Fisher). In brief, beads were first added to a 96 well plate and washed (Biotek ELx405). Samples were then added to the plate containing the mixed antibody-linked beads and incubated at room temperature for 1h followed by overnight incubation at 4°C on a plate shaker (500rpm). Biotinylated detection antibody was then added, after which the plates were incubated at room temperature for 75min on the plate shaker (500rpm). Next, the samples were washed and streptavidin-PE added followed by incubation of the plate 30min at room temperature on the plate shaker (500rpm). The plate was then washed and a reading buffer added to all the wells. Finally, a Luminex Flex 3D instrument was used to read the plates with a lower bound of 50 beads per sample per cytokine. Control assay beads (Radix Biosolutions) were added to all wells. Multiplex ELISA assays were performed in 3 animals from each group. The average cytokine value was taken from 2 separate readings. The fold change in cytokine expression in the transplanted islets treated with pFUS vs. control transplanted islets (i.e. non-treated islets) was calculated using Eq. S1:

$$Fold change vs. control= \frac{\mathrm{OD}_{\mathrm{sample}}- \mathrm{OD}_{\mathrm{control}}}{\mathrm{OD}_{\mathrm{control}}}$$

(**Eq. S1**)

$\mathrm{OD}_{\mathrm{sample}}$: optical density (absorbance) of transplanted islets treated with pFUS; $\mathrm{OD}_{\mathrm{control}}$: optical density (absorbance) of control transplanted islets.

**RESULTS**

Quantification of histological images showed that although there was no significant difference in insulin staining within islets from both experimental groups (percentage of insulin per islet: 49.6±14.3 vs. 50.5±13.6%; P<0.05; **Supplemental Figure 2**), there was a greater degree of vascularity within transplanted islets treated with pFUS. This was confirmed on immunohistochemical analysis which showed a significantly higher expression of vWF in pFUS treated islets (76.1±23.7 vs. 24.2±5.2%; P<0.05; **Supplemental Figure 2**). Transplanted islets treated with pFUS also demonstrated reduced inflammation as evidenced by a reduction in the presence of TNF-α (5.5±0.6 vs. 26.1±11.5%; P<0.05; **Supplemental Figure 2**).


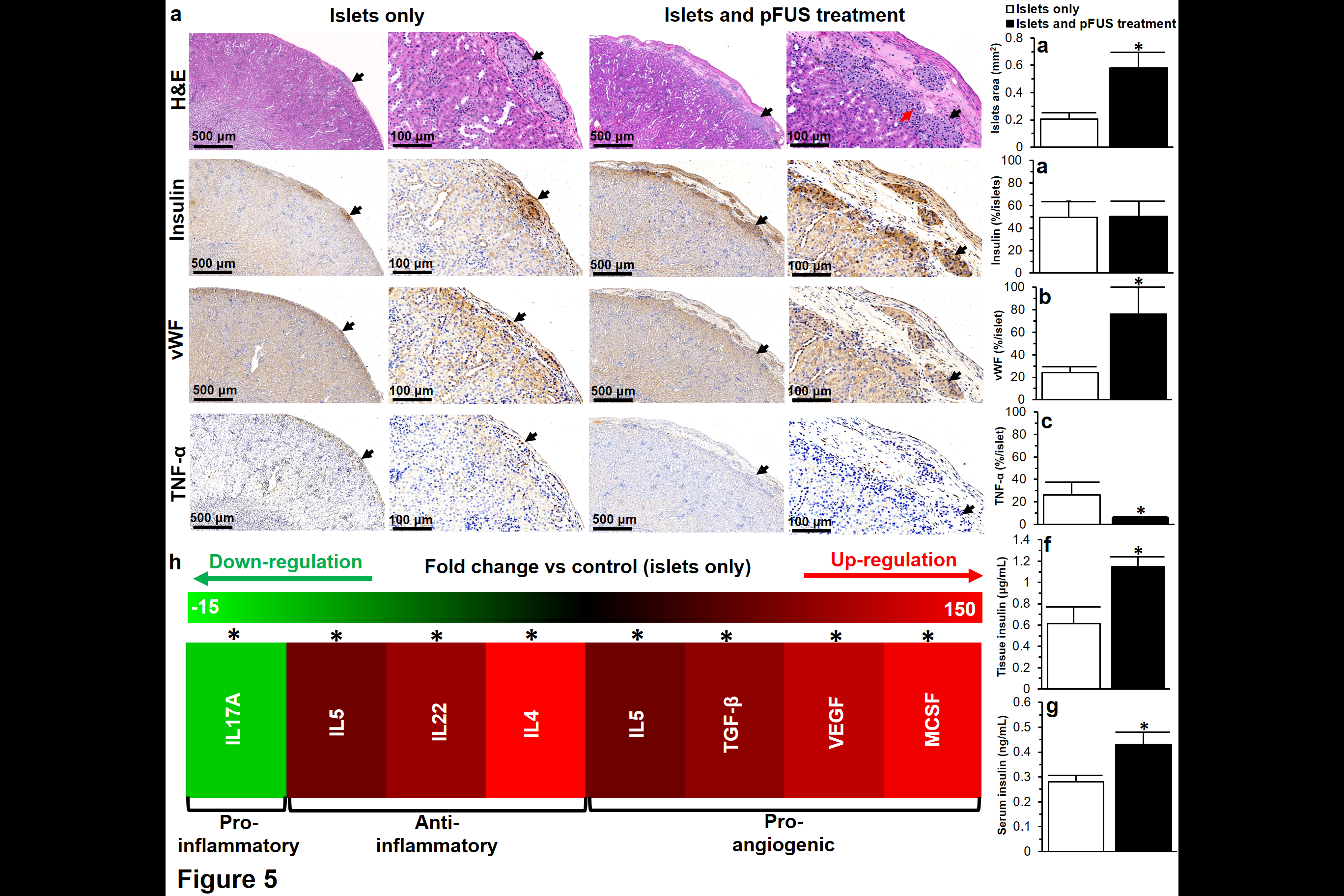


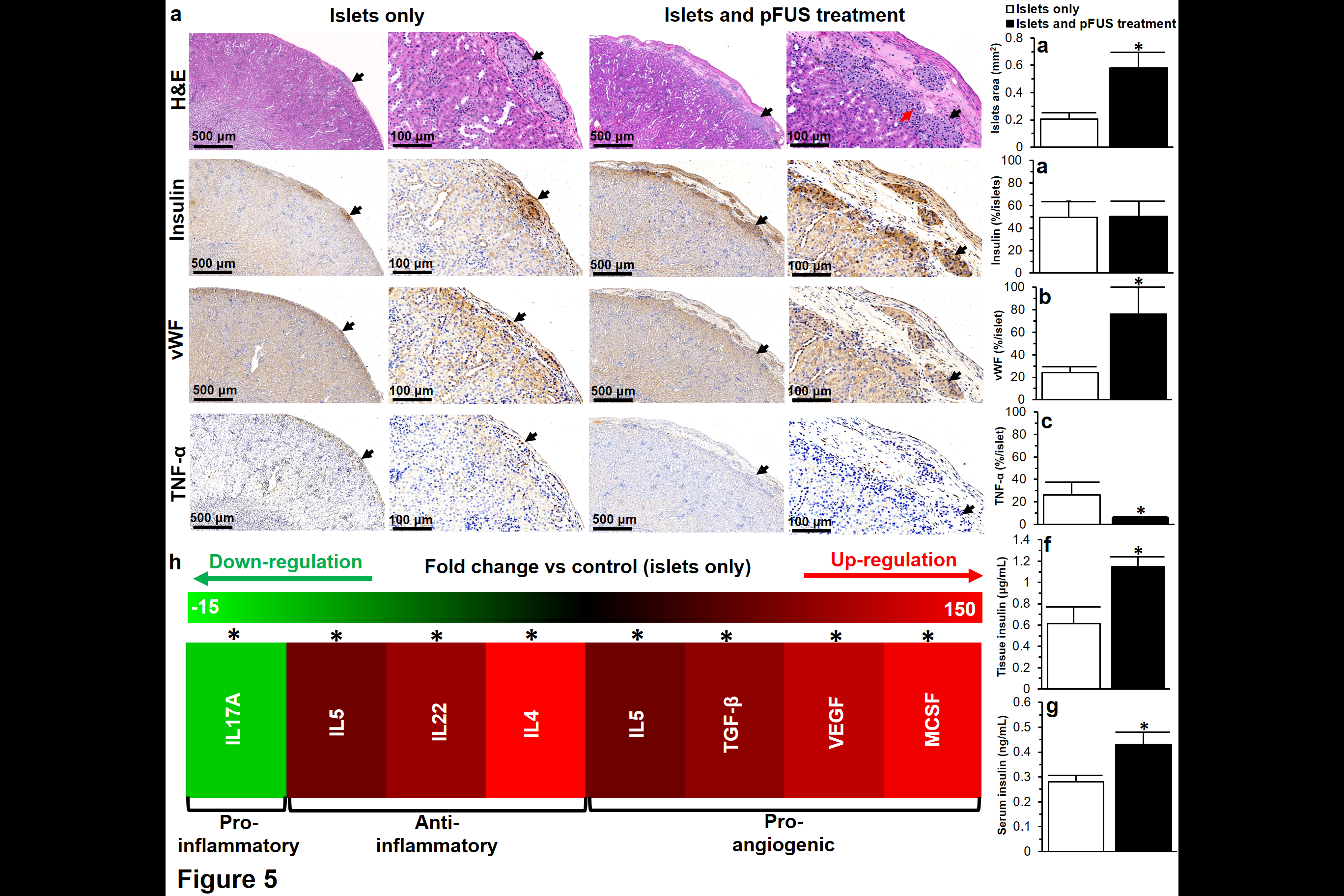


**Supplemental Figure 2:** Quantification of positive (a) insulin, (b) vWF and (c) TNF-α staining within the islets.

Significant differences: *P<0.05 for islets only *vs*. islets treated with pFUS (Student’s unpaired t-test).

**References**

1. Neuman, J. C., Truchan, N. A., Joseph, J. W. & Kimple, M. E. A Method for Mouse Pancreatic Islet Isolation and Intracellular cAMP Determination. *J. Vis. Exp.* e50374 (2014). doi:10.3791/50374

2. Kenty, J. H. R. & Melton, D. A. Testing pancreatic islet function at the single cell level by calcium influx with associated marker expression. *PLoS One* **10**, (2015).

3. Abdulreda, M. *et al.* Measuring dynamic hormone release from pancreatic islets using perifusion assay. *Protoc. Exch.* (2011). doi:10.1038/protex.2011.260

4. Arda, H. E. *et al.* Age-dependent pancreatic gene regulation reveals mechanisms governing human β cell function. *Cell Metab.* **23**, 909–920 (2016).

5. Pfeiffer, T. *et al.* Rapid functional evaluation of beta-cells by extracellular recording of membrane potential oscillations with microelectrode arrays. *Pflugers Arch. Eur. J. Physiol.* **462**, 835–840 (2011).

6. Yang, H. & Wright, J. R. Human β cells are exceedingly resistant to streptozotocin in vivo. *Endocrinology* **143**, 2491–2495 (2002).
